# Spatial niche differentiation and key driving factors of dominant benthic macroinvertebrates in Qinhuangdao City, China

**DOI:** 10.1038/s41598-025-09965-1

**Published:** 2025-07-05

**Authors:** Qingqing Qi, Chunhua He, Xu Liu, Fei Wang, Zezhong Zhang, Kai Feng, Hexin Lai

**Affiliations:** 1 School of Water Conservancy, North China University of Water Resources and Electric Power, Zhengzhou, 450046 China; 2 School of Water and Environment, Chang’an University, Xi’an, 710054 China; 3 Qinhuangdao River and Lake Chief Service Center, Qinhuangdao, 066000 China

**Keywords:** Dominance degree model, Niche, Driving factors, Spatial distribution, Partial correlation analysis, Biodiversity, Ecosystem ecology, Freshwater ecology

## Abstract

Climate change and human activities have led to serious challenges and threats to the global water environment. Protecting suitable benthic niches and clarifying the drivers of niche changes can effectively regulate the intensity of human activities and cope with the impacts of climate change. This study took the sampling data of Qinhuangdao in 2023 as the basis, and used the dominance model to select dominant taxa. Then, it calculated the niche breadth and overlap of the dominant benthic macroinvertebrate using niche models. What’s more, it combined canonical correlation analysis to analyze the correlation between environmental factors and benthic macroinvertebrate density and biomass. Finally, partial correlation analysis was used to identify key driving factors. The results showed that 13 dominant taxa were selected for the dominance model, *Unio douglasiae* and *Orthetrum coerulescens* had the greatest dominance. For water quality physical metrics, all dominant taxa had the highest mean niche breadth along the conductivity gradient (2.51) and the greatest mean niche overlap along the turbidity gradient (4.82). For water chemistry indicators, the niche breadth of dominant taxa was highest along the biochemical oxygen demand gradient and lowest along the hexavalent chromium gradient. Key drivers of niche breadth and overlap spatial differentiation for dominant taxa were chemical oxygen demand for Cr (COD_Cr), biochemical oxygen demand (BOD) and water temperature (WT). High concentrations of COD-Cr and BOD can change the competition and food chain structure among benthic macroinvertebrates, thus affecting the niche breadth and overlap of benthic communities. WT has a direct impact on the physiological and ecological processes of benthic macroinvertebrates. In estuaries and sandy beaches, the key drivers of benthic niche may be organic carbon and chlorophyll α. This study provides a scientific basis for benthic conservation and ecological restoration in Qinhuangdao, and a reference and guidance for similar benthic macroinvertebrates around the globe to cope with climate change, regulate human activities, and enhance biodiversity.

## Introduction

Benthic macroinvertebrate is an essential and important members of aquatic ecosystems globally^1^. Benthic macroinvertebrates are important for the stability and sustainability of aquatic ecosystems, and their involvement in ecological functions and roles is widely recognized and studied^2,3^. First, benthic macroinvertebrates decompose organic matter, promoting its cycling and providing a rich food source and nutrient base for other aquatic organisms, this process helps maintain the balance and sustainable supply of nutrients in the watershed^4,5^. Second, the presence and activities of benthic macroinvertebrate have a positive impact on water quality improvement and substrate protection in aquatic ecosystems^6^. They promote aeration and oxidation at the bottom of the water body by stirring and aerating the substrate, which helps to reduce the accumulation and retention of hazardous substances, and maintains the healthy state of the aquatic ecosystem^7,8^. In addition, benthic macroinvertebrate can adsorb and enrich heavy metals and organic pollutants in the substrate, playing a positive role in purifying the water body and repairing the ecological environment of the water body, which is of great significance to the sustainable use of water resources^9,10^. A comprehensive understanding of the ecological functions and roles of benthic macroinvertebrate contributes to better protecting the ecological environment of the watershed, utilizing its resources, and providing a scientific basis for the healthy development and sustainable utilization of water ecosystems^11,12^.

With the intensification of human utilization of water resources and the growth of environmental pollution, the protection and restoration of the ecological environment have become a hot topic worldwide^13^. As a key component of aquatic ecosystems, the study of the niche characteristics of benthic macroinvertebrate is an important way to understand the functioning of aquatic ecosystems and protect them, which has both theoretical significance and practical application value^1^. Deep exploration of the niche characteristics of benthic macroinvertebrates not only enhances our understanding of their pivotal roles and interrelationships within watershed ecosystems but also facilitates strategies for sustainable management^14^.

In benthic niche studies, the analysis of driving factors is crucial^15^. Driving factors are factors that influence the distribution of benthic macroinvertebrates and the ecological functioning, primarily including temperature, nutritional components and hydrological elements^16,17^. Changes in these elements can directly affect the survival, reproduction, and distribution of benthic macroinvertebrate in aquatic ecosystems, and thus have important implications for the stability of the entire ecosystem^18^. Zhang, et al.^19^ in the Weihe River watershed of China showed that nitrate (NH_3_-N), turbidity (Tub), dissolved oxygen (DO) in water environment and heavy metals in sediment were the main environmental factors affecting the community structure of benthic macroinvertebrate. Through in-depth exploration of the driving elements to which benthic macroinvertebrates are subjected and their interactions, it can provide an important basis for predicting and explaining ecological dynamic changes in watershed ecosystems^20^. Therefore, analyzing the role of benthic niche driving elements from multiple perspectives is of great significance for revealing the operation law of the watershed ecosystem and protecting it^21,22^.

Domestic research on the niches of benthic macroinvertebrate communities has been carried out in areas such as streams, rivers and lakes such as Jinsha River, Nenjiang River and Laurentian Great Lakes^23–25^. The research finds that the more niche resources that benthic macroinvertebrate in plain streams can utilize, the less competition the biological community will have for resources^26^. The moderate lateral hydrological connectivity of plateau rivers provides more niches for benthic macroinvertebrate, which is conducive to increasing taxa diversity^27^. Domestic scholars have revealed the trophic level relationships among taxa through stable isotope analysis and explored the dynamic laws of niche width and overlap in combination with habitat heterogeneity. For instance, studies on benthic macroinvertebrate in estuarine areas have shown that salinity gradient and sediment type are the key factors driving niche differentiation^28^. International scholars pay more attention to the construction of multi-dimensional niche models and cross-scale integration. Based on the taxa identification method of eDNA technology and metabarcoding, combined with machine learning algorithms, the precise characterization of the niches of benthic macroinvertebrate in extreme environments such as deep sea and polar regions has been achieved^29–31^. However, the research on the niche differentiation mechanism of benthic macroinvertebrate in the transition zones of brackish and fresh water such as estuaries and coastal waters is still relatively weak, especially lacking systematic analysis of complex habitat gradients such as drastic changes in salinity, tidal cycles and biological interactions.

Qinhuangdao, as a typical area of the Bohai Bay, features a semi-enclosed bay, the convergence of fresh and salt water, diverse substrates, and intense human activities, making it of great significance for the study of coastal ecological environment^32,33^. However, little has been done to analyze the elements driving the spatial differentiation of benthic niche in Qinhuangdao. Consequently, this paper aims to study the law of spatial differentiation of niche and key driving factors of dominant benthic niche in Qinhuangdao, with the following specific objectives: (1) identify dominant benthic taxa in Qinhuangdao; (2) analyze the law of spatial differentiation of niches of dominant taxa; and (3) identify the key driving factors of spatio-temporal differentiation of niches of benthic macroinvertebrates. Through these studies, an in-depth study of the niche spatial differentiation patterns and key driving factors of dominant taxa can help reveal the interactions among taxa, resource utilization patterns, and environmental adaptation strategies, and thus promote the development of niche theory. At the same time, this study can provide a reference for ecosystem management and conservation in other similar areas and promote the process of global ecosystem conservation.

## Study area and data collection

### Study area

**Fig. 1:**
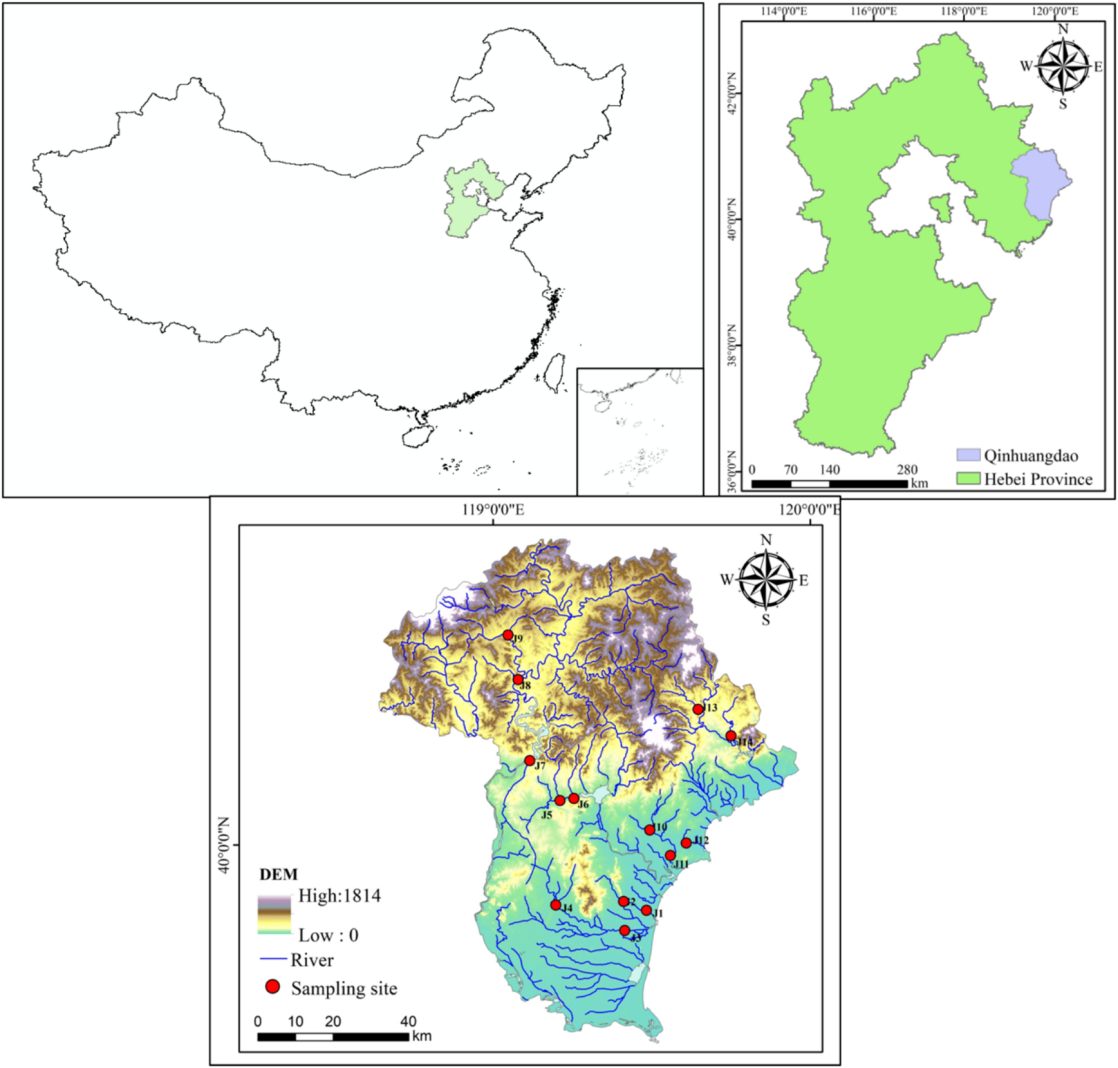
Distribution map of 14 sampling points in Qinhuangdao. Map was created by ArcMap 10.6 (https://www.arcgis.net.cn/download.html), and the data set is provided by Geographic remote sensing ecological network platform (www.gisrs.cn).

The study area is located in Qinhuangdao in the northeast of Hebei Province, with geographic coordinates ranging from 39°24′ to 40°37′ N latitude and 118°33′ to 119°51′ E longitude, and a land area of 7802km^2^^34^. Qinhuangdao belongs to the Luanhe River system and the east Hebei coastal water system^34^. 13 rivers are entering the sea, including Luanhe River, Yinma River, Dongsha River, Renzao River, Yanghe River, Daihe River, Xinhe River, Tanghe River, Xinkai River, Paihong River, Shahe River, Qili Lake and Shihe River^35^. Qinhuangdao has a continental monsoon climate with four distinct seasons, because of its proximity to the ocean and its strong influence from the sea, the climate is relatively mild, with little rain and dryness in spring, warm summers without scorching heat, cool and sunny autumns, and long winters without severe cold^36^. The average annual temperature is 11.9 ℃, with a maximum temperature of 40.2℃ and a minimum temperature of −21.0 ℃^37^. The average annual precipitation is 736.3 mm, and the seasonal distribution of precipitation is uneven, mostly concentrated in summer, and the precipitation from June to August can reach 70% ~ 80% of the total annual precipitation^34^. A total of seven rivers, including Dongsha River, Yinma River, Xiyang River, Qinglong River, Daihe River, Xinhe River, and Shihe River in Qinhuangdao were studied. The locations of the river sampling sections are shown in Fig. 1.

### Data

Field research data were used in this study, and the sampling period was from June 18 to 24, 2023, sampling points were arranged in accordance with the national standard^13^ and “Technical Outline for the Evaluation of the Health of Rivers and Lakes in Hebei Province (Trial)”. A total of 14 sampling points were set up for this sampling, and the distribution of the points is shown in Fig. 1. Sampling using the Peterson mud collector model ETC-200 [1/(16 m^2^)], each sampling point was collected with 3 parallel samples, and the sampling thickness was generally 10-15 cm, the collected sediment samples were placed in a sieve for cleaning and then sorted. According to the national standard^13^, the samples to be screened need to be placed on a 40-mesh (0.793 mm per well) screen for screening and sorting. After the samples were brought back to the laboratory, they were fixed and stored for a long time with 70% alcohol or 5% formalin solution, and all individuals were identified to reliable classification units by Hebei Institute of Environmental Engineering. Collected samples were morphologically identified using standard taxonomic references, with the goal of reaching species-level classification. Specimens were preserved for all identified samples.

Hydrological and water quality data (18 physical and chemical indicators) were obtained from the official website of the Qinhuangdao Ecological Environment Bureau. Analyzed variables included:

Physical data: water temperature (WT), turbidity (Turb), pH and conductivity (Cond);

Chemical data: dissolved oxygen (DO), permanganate (COD_Mn), chemical oxygen demand (COD_Cr), ammonia nitrogen (NH₄-N), total phosphorus (TP), total nitrogen (TN), biochemical oxygen demand (BOD), fluoride (F⁻), nitrate (NO₃-N), nitrite (NO₂-N), sulphide (S^2^⁻), copper (Cu), zinc (Zn), hexavalent chromium (Cr⁶⁺).

## Methods

### Dominance model

The sampling was carried out to obtain a wider variety of benthic taxa. Given the differences in density and biomass of different benthic macroinvertebrates, it was necessary to screen the samples and select more numerous and representative taxa to be studied in order to reflect the characteristics of the whole community, which are referred to as dominant taxa^7^. Zhao, et al.^38^ proposed a dominance degree model based on the density and biomass of taxa. By analyzing these factors, researchers can better understand taxa interrelationships, competition dynamics, and overall ecosystem functioning. The advantage degree calculation is carried out in Excel using the following formula:

1 $${I}_{mportance,i}={\omega }_{1}{P}_{a,i}+{\omega }_{2}{P}_{b,i}$$

where ${I}_{mportance}$ represents the dominance of a taxa; ${P}_{a}$ and ${P}_{b}$ respectively considering taxa space/missing taxa density and the ratio of biomass and total biomass, $${P}_{a,i}=\frac{{N}_{i}}{\sum \begin{array}{c}N\\ i\end{array}}$$, $${P}_{b,i}=\frac{{B}_{i}}{\sum \begin{array}{c}B\\ i\end{array}}$$, ${N}_{i}$ is the density of the i taxa and ${B}_{i}$ is the biomass of the i taxa, ${\omega }_{1}$ and ${\omega }_{2}$ are the weights of density and biomass, $\frac{{\omega }_{1}}{{\omega }_{2}}=\frac{a}{b}$, ${\omega }_{1}$+${\omega }_{2}$=1.0, *a、b* refer to the reference for the determination formula^39^.

Due to the large difference in individual biomass between *Mollusca* and other taxa, in order to reduce the impact of the gap, *Mollusca* will be screened separately for dominant taxa.

After calculating the degree of dominance of all taxa, the curvature method was used to determine the dominant taxa. The curvature method is simple and easy to understand, and by visualizing the cumulative dominance curve, the difference in dominance increment between before and after the breakpoint can be directly observed, and the dominant taxa can be determined based on the difference^39,40^. The calculation formula of the curvature method is as follows:

2 $$k=\frac{\frac{{d}^{2}y}{d{x}^{2}}}{{\left[1+{(\frac{dy}{dx})}^{2}\right]}^{3/2}}$$

where *k* is curvature, $\frac{dy}{dx}$ is the derivative of y with respect to x, and $\frac{{d}^{2}y}{d{x}^{2}}$ is the derivative of $\frac{dy}{dx}$ with respect to x again.

### Niche models

Levins’ niche breadth model^13^ is a model used to describe the activities of multiple taxa in an ecosystem and their resource utilization. Levins’ niche breadth model accounts for species interactions and competition, offering a comprehensive framework to analyze species distribution and resource utilization within ecosystems. Its adaptability makes it suitable for modeling complex ecological dynamics. Additionally, the model employs a simple mathematical formulation to describe species-resource relationships, which is easy to understand and apply. The niche breadth can be calculated by using DPS software (v9.50). Levins’ niche breadth model is as follows:

3 $${\text{B}}_{\text{i}}=\frac{1}{\sum_{\text{j}=1}^{\text{R}}{{\text{P}}_{\text{ij}}}^{2}}$$

where ${B}_{i}$ is the niche breadth of taxa *I*; ${P}_{ij}$ represents the proportion of the quantity of taxa *i* on the resource gradient *j* to the total quantity of the taxa; *R* refers to the quantity of resource gradients, which are classified in accordance with the environmental quality standards for surface water in China^13^.

Pianka’s niche overlap model^41^ quantifies interspecific competition and resource-use overlap, revealing species coexistence mechanisms and resource partitioning strategies. This approach measures both the degree of resource utilization overlap and the intensity of competition, providing insights into species interactions. We can realize niche overlap with the DPS software (v9.50). Pianka’s niche overlap model is as follows:

4 $${\text{O}}_{\text{ik}}=\frac{\sum_{\text{i}=1}^{\text{n}}{\text{P}}_{\text{ij}}{\text{P}}_{\text{ik}}}{\sqrt{\sum_{\text{i}=1}^{\text{n}}{\text{P}}_{\text{ij}}^{2}\sum_{\text{i}=1}^{\text{n}}{\text{P}}_{\text{ik}}^{2}}}$$

where ${O}_{ik}$ is the niche overlap of taxa *i* on taxa *k*; ${P}_{ij}$ and ${P}_{ik}$ represent the proportion of the number of taxa *i* and taxa *k* on resource gradient *j* to the total number of taxa *i* and *k*, ${O}_{ik}$≠${O}_{ki}$.

### Canonical correlation analysis

Canonical Correlation Analysis (CCA) is a multivariate statistical analysis method for exploring the correlation between multiple groups of variables^13,42^. CCA helps researchers reveal correlations among variables in different groups, identify their most relevant linear combinations, and reduce dataset dimensionality to lower analysis complexity. CCA calculations can now be implemented using Canoco5 software (v1.36), the calculation formula of CCA is shown in Eq. (5).

5 $$maxcorr\left(U,V\right)=\frac{{a}^{T}\sum_{12}b}{{({a}^{T}\sum_{11}a\times {b}^{T}\sum_{22}b)}^{1/2}}$$

where $maxcorr\left(U,V\right)$ is the maximum correlation coefficient between *U* and *V*, *U* and *V* are linear expressions of variables *X* and *Y* respectively, $U={a}_{1}{x}_{1}+{a}_{2}{x}_{2}+\cdots +{a}_{P}{x}_{P}={a}^{T}X$, $V={b}_{1}{y}_{1}+{b}_{2}{y}_{2}+\cdots +{b}_{q}{y}_{q}={b}^{T}Y$, *p* is the number of eigenvalues of variable *X*, and *q* is the number of eigenvalues of variable *Y*, $\sum 11$ and $\sum 22$ are the elements of the covariance matrix of variables *X* and *Y*.

### Partial correlations analysis

Partial Correlations Analysis (also named net correlation analysis) is a statistical method that assesses relationships between two variables while controlling for the influence of others, thereby improving correlation accuracy^43,44^. In this paper, Partial Least Squares Regression (PLSR) model^45,46^ is adopted, which is a linear regression analysis method based on least squares method. The regression model is established after dimensionality reduction of independent variables and dependent variables. PLS-VIP (Variable Importance in Projection, VIP) comprehensively reflects the influence of variables on the predictive ability of the overall model by accumulating their contribution degrees on all latent variables, and it is insensitive to multicollinearity and has strong result stability^13,47^. The BETA value is mainly used to explain the effect of the variable on the response direction (positive/negative correlation), and does not apply to the ranking of importance^48^. In PLS analysis, both can be used for variable screening, among which PLS-VIP has become the most commonly used indicator due to the above advantages. We usually calculate the VIP value of the *J*^*th*^ variable using Python 3.10, as follows:

6 $$VIP_{j} = \sqrt {\frac{{P\mathop \sum \nolimits_{k = 1}^{h} \left( {c_{k}^{2} t_{k}^{\prime } t_{k} } \right)\left( {w_{jk} } \right)^{2} }}{{\mathop \sum \nolimits_{k = 1}^{h} c_{k}^{2} t_{k}{\prime} t_{k} }}}$$

where *p* means that there are initially *p* variables involved in the analysis; *h* means that a total of *h* iterations were performed (a total of h dimensions were obtained); ${w}_{jk}$ represents the weight used when variable *j* is mapped at the *kth* iteration, it reflects the interpretation degree of variable *j* to the *kth* mapping result ${X}_{k}$; $c_{k}^{2} t_{k} t_{k}$ indicates the degree to which ${Y}_{k}$ is interpreted as the result of the *kth* mapping ${X}_{k}$ (note: ${X}_{k}$ is the *kth* mapping of the independent variable *X*, and ${Y}_{k}$ is the part of the dependent variable *Y* that is interpreted at the *kth* iteration).

## Results

### Identification of dominant taxa

A total of 40 taxa of benthic macroinvertebrates were identified, belonging to 4 phylums, 7 classis, 28 families and 36 genus. All taxa names have been verified in the “Catalogue of Life China: 2024 Annual Checklist” (http://www.sp2000.org.cn) to ensure consistency with the latest taxonomic revision, as shown in Table 1.

**Table 1:** Density and biomass of benthic macroinvertebrates in seven rivers of Qinhuangdao.

Phylum	Classis	Ordo	Familia	Genus	Species	Density (ind/m^2^)	Biomass (g/m^2^)
Platyhelminthes	Planarians					7.7	0.11
Annelida	Oligochaeta	Oligochaeta	Tubificidae	Monopylephorus		167.8	1.50
			Naididae	Nais		9.80	0.10
				Hydroides ezoensir		76.6	0.54
		Enchytraeida	Enchytraeidae	Enchytraeus		19.16	0.09
		Haplotaxida	Megascolecidae		Branchiura sowerbyi	20.82	0.67
		Plesiopora	Tubificidae	Limnodrilus		34.33	0.17
				Tubifex		20.45	0.34
	Hirudinea	Rhynchobdellida	Glossiphonidae	Glossiphonia		21.08	0.443
					Tranquilitatiszeae	7.70	0.54
		Hirudinida	Herpobdellidae		Erpobdella	11.52	0.08
Arthropoda	Insecta	Odonata	Gomphidae		Orthetrum coerulescens	5.28	4.40
			Aeshnidae		Larva Ephemeropterae	15.33	7.82
			Coenagrionidae			15.33	0.565
			Calopterygidae			23.00	1.23
		Diptera	Chironomidae		Aedes albopictus	114.02	0.53
					Chironomus	164.39	1.31
					Chironomid	61.30	0.39
			Ceratopogonidae	Culicoides Latreille		7.66	0.08
		Rhyngota	Belostomatidae			11.50	0.46
		Ephemeroptera	Ephemeridae	Ephemera		32.85	2.12
			Baetidae		Baetis	7.66	0.08
	Malacostraca	Decapoda	Palaemonoidea	Palaemonidae		70.78	3.58
		Amphipoda	Gammaridea			53.60	1.18
Mollusca	Gastropoda	Mesogastropoda	Viviparidae	Bellamya		36.88	43.31
				Cipangopaludina		81.17	66.53
			Planorbidae		Valvata	7.94	2.77
			Pomatiopsidae	Lymnaea		14.35	3.96
		Basommatophora	Lymnaeidae	Radix		128.88	24.56
				Lymnaeidae		23.21	1.16
				Galba		12.43	0.44
			Planorbidae	Hippeutis		129.79	1.53
				Gyraulus		22.03	0.42
			Physidae	Physa		15.30	0.61
	Lamellibranchia	Veneroida	Corbiculidae	Corbicula		22.75	9.43
					Sphaeriidae	19.168	2.45
				Pisidium amnicum		15.33	0.54
		Unionoida	Unionidae	Anodonta		9.90	1.38
					Unio douglasiae	11.50	350.98
					Anodonta woodianawoodiana	7.66	0.31

In Table 1, different groups show significant differences in the ecosystem. The *Annelida* is mainly composed of *Oligochaeta*, with the highest density proportion, the *Genus Monopylephorus* of the *Family Tubificidae* reaches 167.8 ind/m^2^, but the biomass is relatively low, only 1.50 g/m^2^. Among the *Arthropoda*, the *Family Chironomidae* of the *Order Diptera* and the *Order Decapoda* of the *Class Malacostraca* have the upper hand, such as the density of the *Family Chironomus* is 164.39 ind/m^2^ and the biomass of the *Family Palaemonoidea* is 3.58 g/m^2^. While the biomass of the larvae of the *Order Odonata* of the *Class Insecta* stands out, with the biomass of the *Orthetrum coerulescens* is 7.82 g/m^2^. The *Mollusca* phylum holds an absolute advantage in biomass, especially the genera of the *Cipangopaludina* (66.53 g/m^2^) and the *Bellamya* (43.31 g/m^2^), as well as the *Unio douglasiae* (350.98 g/m^2^). The density and biomass of the *Class Planarians* and the *Class Hirudinea* are both relatively low, and their ecological functions are concentrated in microhabitat regulation.

The water quality characteristics of the seven rivers are summarized in Table 2, covering 18 physical and chemical indicators.

**Table 2:** Water quality indicators of 7 rivers sampled in Qinhuangdao.

Habitat environment	Abbreviation	Name	Unit	Range	SD
Physical	WT	Water temperature	℃	20.50–25.80	1.41
	Turb	Turbidity	NTU	3.50–45.50	12.74
	pH			7.43–8.69	0.53
	Cond	Conductivity	ms/m	36.61–3810.23	1172.86
Chemical	DO	Dissolved oxygen	mg/L	4.93–12.74	2.31
	COD_Mn	Permanganate		1.74–6.90	1.33
	COD_Cr	Chemical oxygen demand for Cr		2.43–18.74	6.05
	NH_4_-N	Ammonia nitrogen		0.05–1.38	0.34
	TP	Total phosphorus		0.02–0.33	0.08
	TN	Total nitrogen		0.69–6.12	1.49
	BOD	Biochemical oxygen demand		0.03–24.05	7.65
	F-	Fluoride		0.19–1.09	0.23
	NO_3_-N	Nitrate		1.06–11.48	2.49
	NO_2_-N	Nitrite		0.01–0.49	0.13
	S^2-^	Sulphide		0.0020–0.0050	0.0015
	Cu	Copper		0.0005–0.0030	0.0006
	Zn	Zinc		0.0007–0.0500	0.0245
	Cr⁶⁺	Hexavalent chromium		0.0020–0.0040	0.0009

The unit of water quality chemical index is the same as DO, which is mg/L. SD is the standard deviation.

**Fig. 2:**
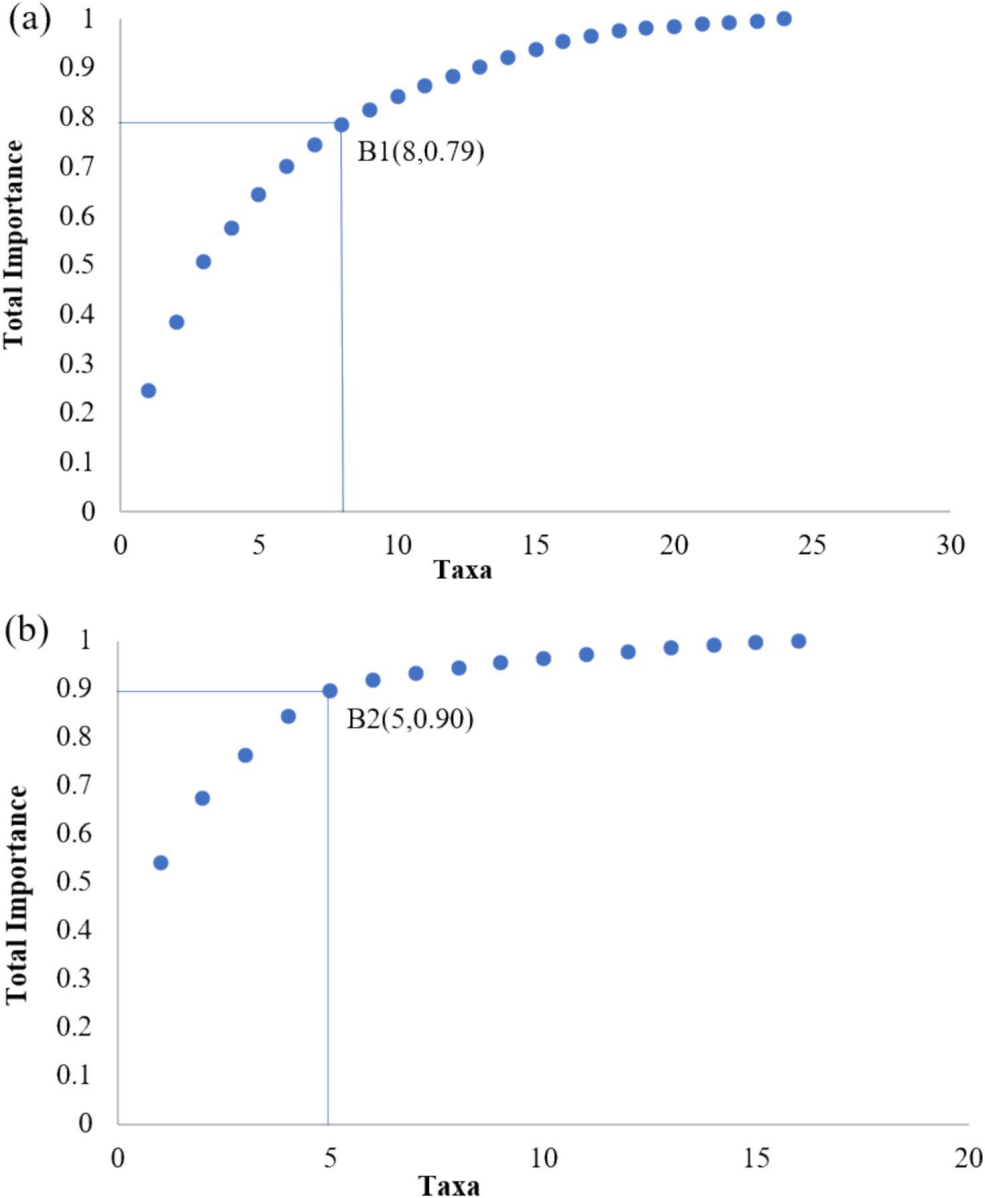
Cumulative dominance gradient curves for all of *Platyhelminthes*, *Annelida* and *Arthropoda* (**a**), *Molluscas* (**b**), with B1 and B2 being the inflection points.

Based on Eq. (1) and the weight determination method in the literature^39^, the weighting factors for density (ω₁₁) and biomass (ω₁₂) were determined as follows: for *Platyhelminthes*, *Annelida* and *Arthropoda*, ${\omega }_{11}$ was 0.42 and ${\omega }_{12}$ was 0.58, for *Mollusca*, ${\omega }_{21}$ was 0.22 and ${\omega }_{22}$ was 0.78. The importance ranking of each taxonomic group is shown in Table 3 (Eq. 1), and the cumulative importance curve is shown in Fig. 2. The groups before the inflection point of the cumulative dominance curve graph are the dominant groups (Eq. 2).

**Table 3:** Importance of benthic macroinvertebrate taxa.

Platyhelminthes, Annelida, Arthropoda			Mollusca		
Serial	Taxa	Importance	Serial	Taxa	Importance
1	Orthetrum coerulescens	0.25	1	Unio douglasiae	0.54
2	Larva Ephemeropterae	0.14	2	Cipangopaludina	0.13
3	Palaemonidae	0.12	3	Radix	0.09
4	Ephemera	0.07	4	Bellamya	0.08
5	Monopylephorus	0.07	5	Hippeutis	0.05
6	Chironomus	0.06	6	Corbicula	0.02
7	Aedes albopictus	0.04	7	Lymnaea	0.01
8	Branchiura sowerbyi	0.04	8	Sphaeriidae	0.01
9	Calopterygidae	0.03	9	Lymnaeidae	0.01
10	Hydroides ezoensir	0.03	10	Gyraulus	0.01
11	Gammaridea	0.02	11	Valvata	0.01
12	Coenagrionidae	0.02	12	Physa	0.01
13	Chironomid	0.02	13	Pisidium amnicum	0.01
14	Tranquilitatiszeae	0.02	14	Anodonta	0.01
15	Glossiphonia	0.02	15	Galba	0.01
16	Belostomatidae	0.02	16	Anodonta woodianawoodiana	0.00
17	Tubifex	0.01			
18	Limnodrilus	0.01			
19	Enchytraeus	0.00			
20	Planarians	0.00			
21	Nais	0.00			
22	Erpobdella	0.00			
23	Culicoides Latreille	0.00			
24	Baetis	0.00

In Fig. 2, the cumulative dominance of species rose rapidly but the growth rate slowed down before the inflection point, indicating that species before the change in the upward trend were more important in the benthic biological community. Therefore, the first 5 taxa of *Mollusca* in Table 2 are the dominant taxa, corresponding to the taxa of *Unio douglasiae* (0.54), *Cipangopaludina* sp*.* (0.13), *Radix* sp*.* (0.09), *Bellamya* sp*.* (0.08), *Hippeutis* sp*.* (0.05). In Table 2, the first 8 taxa of *Platyhelminthes*, *Annelida* and *Arthropoda* are the dominant taxa, corresponding to the taxa of *Orthetrum coerulescens* (0.25), *Larva Ephemeropterae* (0.14), *Palaemonidae* (0.12), *Ephemera* (0.07), *Monopylephorus* sp. (0.07), *Chironomus* (0.06), *Aedes albopictus* (0.04), and *Branchiura sowerbyi* (0.04).

### Dominant taxa niche along water quality factors

#### Niche breadth and overlap of dominant taxa along water quality physical indexes

The niche breadths and overlaps of 13 dominant taxa calculated along the gradients of 4 water quality physical indexes are shown in Tables 4 and 5. In Table 4, among the niches calculated along the gradient of physical indicators of water quality, *Chironomus* sp. had the highest average niche breadth (2.34), and *Unio douglasiae* and *Orthetrum coerulescens* had the lowest average niche breadth (1.00). All the dominant taxa had the highest average niche breadth along the Cond gradient (2.51) and the lowest niche breadth along the pH gradient (1.54).

**Table 4:** Niche breadth of dominant taxa along the gradient of water quality physical index.

Name	Cond	WT	pH	Turb	Mean	Rank
Orthetrum coerulescens	1.00	1.00	1.00	1.00	1.00	13
Larva Ephemeropterae	2.39	2.04	1.64	2.04	2.03	5
Palaemonidae	2.36	3.48	1.28	1.47	2.15	2
Ephemera	2.21	2.02	2.75	1.54	2.13	3
Monopylephorus	1.30	1.08	1.29	1.26	1.24	10
Chironomus	1.00	1.00	1.00	1.00	1.00	12
Aedes albopictus	1.66	1.00	1.66	1.00	1.33	9
Branchiura sowerbyi	2.20	2.20	2.20	1.92	2.13	4
Unio douglasiae	2.07	1.92	1.30	1.99	1.82	7
Cipangopaludina	1.06	1.22	1.20	1.07	1.14	11
Radix	2.77	2.23	1.50	2.86	2.34	1
Bellamya	1.93	1.63	1.69	1.35	1.65	8
Hippeutis	2.51	2.16	1.52	1.78	1.99	6
Mean	1.88	1.77	1.54	1.56		
Rank	1	2	4	3

**Table 5:** Niche breadth overlap of dominant taxa under the influence of water quality physical index.

Name	Cond	WT	pH	Turb	Mean	Rank
Orthetrum coerulescens	3.00	3.08	2.63	3.74	3.11	12
Larva Ephemeropterae	3.03	4.24	3.09	4.05	3.60	10
Palaemonidae	3.01	3.77	3.08	3.93	3.45	11
Ephemera	3.98	4.19	3.79	4.18	4.04	7
Monopylephorus	3.30	3.04	3.00	2.61	2.99	13
Chironomus	6.08	2.30	5.94	5.84	5.04	5
Aedes albopictus	4.41	4.68	6.12	3.03	4.56	6
Branchiura sowerbyi	5.64	2.62	1.72	5.35	3.83	9
Unio douglasiae	6.34	5.09	6.11	6.41	5.99	1
Cipangopaludina	6.10	2.46	1.65	5.87	4.02	8
Radix	5.39	5.21	6.08	6.18	5.71	3
Bellamya	6.59	5.18	5.98	6.21	5.99	2
Hippeutis	4.53	4.86	5.93	5.26	5.15	4
Mean	4.72	3.90	4.24	4.82		
Rank	2	4	3	1		

In Table 5, *Ephemera* and *Aedes albopictus* have the highest average niche overlap value (5.99) along the gradient of water quality physical indexes, and *Hippeutis* sp. has the lowest niche overlap (2.99). The average niche overlap of all dominant taxa along the Turb gradient was the highest (4.82), and the average niche overlap of all dominant taxa along the WT gradient was the lowest (3.90).

#### Niche breadth and overlap of dominant taxa under water quality chemical indexes

**Fig. 3:**
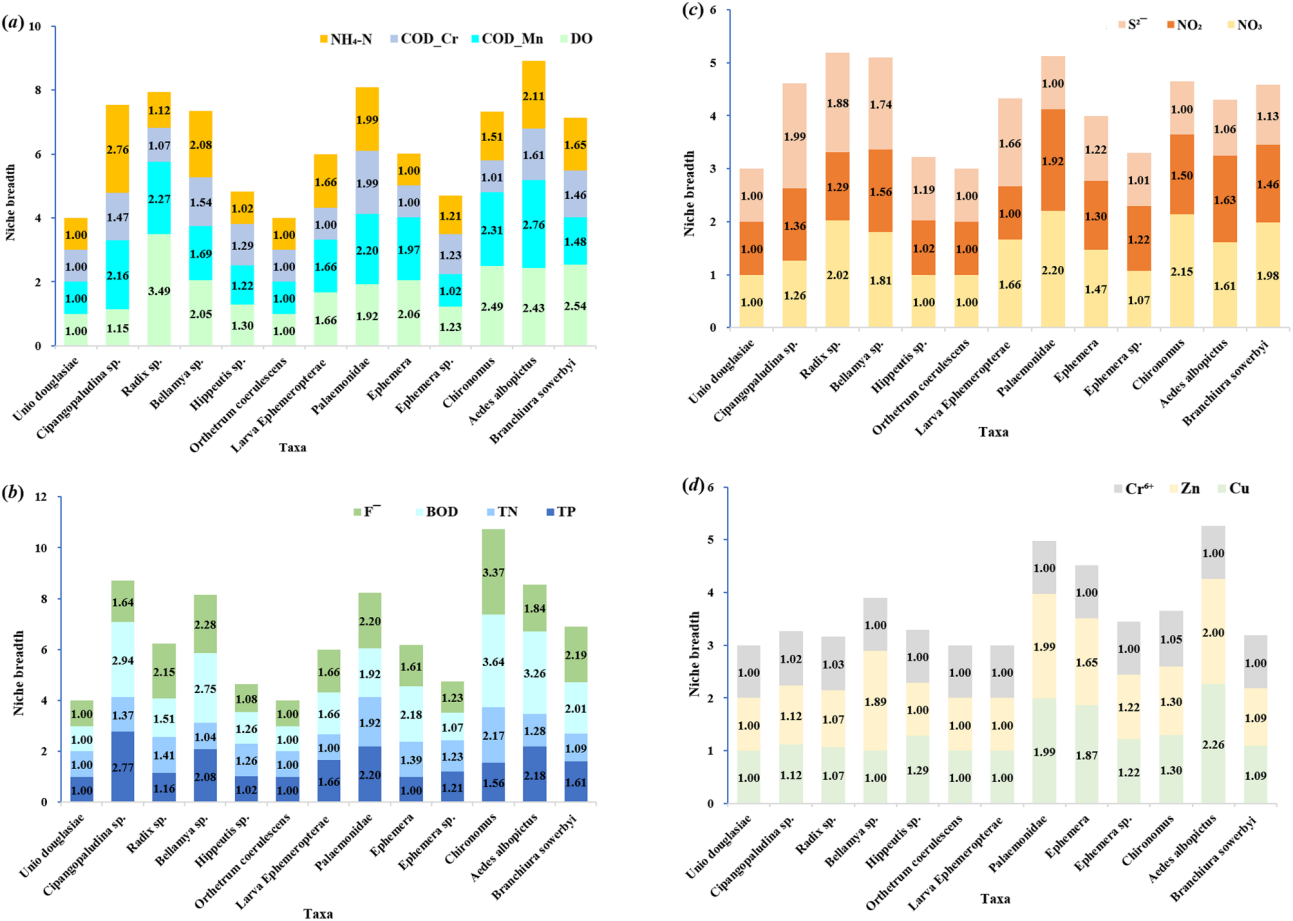
Niche breadth of dominant taxa along the gradient of water quality chemical index, namely (**a**) ammonia nitrogen (NH_4_-N), chemical oxygen demand for Cr (COD_Cr), permanganate (COD_Mn), dissolved oxygen (DO), (**b**) fluoride (F^-^), biochemical oxygen demand (BOD), total nitrogen (TN), total phosphorus (TP), (**c**) sulphide (S^2-^), NO_2_-N, NO_3_-N, (**d**) hexavalent chromium (Cr⁶⁺), Zn, Cu.

The Niche breadth of dominant taxa along the gradient of water quality chemical index is shown in Fig. 3. In Fig. 3, *Aedes albopictus* had the highest average niche breadth along the water quality chemical index gradient (1.93), and had the highest niche breadth along the BOD gradient (3.26) with the lowest niche breadth along the Cr⁶⁺ gradient (1.00). The average niche breadths of *Unio douglasiae* and *Orthetrum coerulescens* were the lowest (1.00) along the water quality chemical index gradient, and the niche breadths of the two dominant taxa were 1.00 along all the chemical index gradient. The average niche breadth of all the dominant taxa was the highest along the BOD gradient (2.02) and the lowest along the Cr⁶⁺ gradient (1.01).

**Fig. 4:**
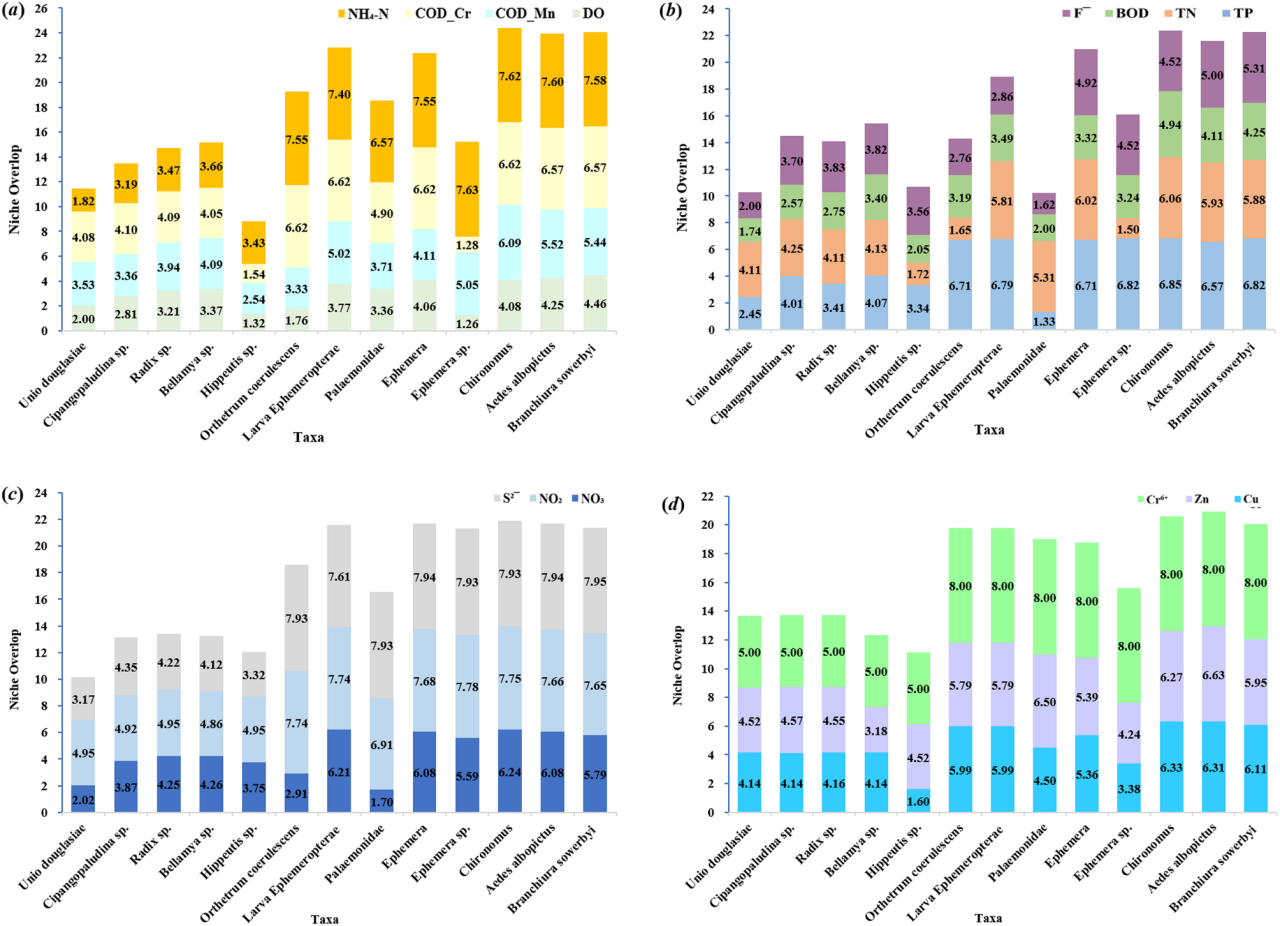
Niche overlap of dominant taxa along the gradient of water quality chemical index, namely (**a**) ammonia nitrogen (NH_4_-N), chemical oxygen demand for Cr (COD_Cr), permanganate (COD_Mn), dissolved oxygen (DO), (**b**) fluoride (F^-^), biochemical oxygen demand (BOD), total nitrogen (TN), total phosphorus (TP), (**c**) sulphide (S^2-^), NO_2_-N, NO_3_-N, (**d**) hexavalent chromium (Cr⁶⁺), Zn, Cu.

The niche overlap of dominant taxa along the gradient of water quality chemical indexes is shown in Fig. 4. In Fig. 4, *Chironomus* has the highest average niche overlap (6.38) along the water quality chemical index gradient, followed by *Aedes albopictus* and *Branchiura sowerbyi*. The average niche overlap of these two dominant taxa is 6.30 and 6.27, respectively. The average niche overlap of these three dominant taxa is much higher than that of other dominant taxa. *Chironomus* has the highest niche overlap along the Cr⁶⁺ gradient (8.00) and the lowest niche overlap along the DO gradient (4.04). The average niche overlap of all dominant taxa along the Cr⁶⁺ gradient was the highest (6.85), and the average niche overlap along the DO gradient was the lowest (3.05).

#### Spatial evolution of niche breadth and overlap

The niche breadths and overlaps of all dominant taxa at different sampling sites along the gradients of water quality physical indexes are shown in Table 6. In Table 6, the maximum average niche breadth of the dominant taxa in the northern mountainous area along the gradient of water quality physical index was 1.97, while the mean value in the eastern coastal area was the lowest (1.80). The maximum niche overlap value in the northern mountainous area was 5.26 and the minimum value was 4.35, which was the area with the highest mean value (4.81). The central region has the area with the lowest mean niche overlap value (4.31).

**Table 6:** Distribution table of dominant taxa along gradient niche breadth and overlapping space of water quality physical index.

River	Section	District	Niche breadth	Niche overlap
Dongsha river	J1	Southern coastal area	1.99	4.22
	J2		1.81	4.53
Yinma river	J3		1.90	4.43
	J4		1.82	5.03
Xiyang river	J5	Central region	1.89	4.20
	J6		1.95	4.92
Qinglong river	J7		1.51	3.48
	J8	Northern mountain area	1.94	4.35
	J9		1.99	5.26
Daihe river	J10	Central region	2.05	4.62
	J11	Eastern coastal area	1.60	4.08
Xinhe river	J12		1.92	4.30
Shihe river	J13		1.86	4.55
	J14		1.82	5.22

The niche breadth and overlap of dominant taxa at different sampling sites along the gradient of water quality chemical indexes are shown in Table 7. In Table 7, the maximum average niche breadth of the dominant taxa in the northern mountainous area along the gradient of water quality physical index was 1.72, while the mean value in the eastern coastal area was the lowest (1.56). The average niche breadth in the central region is close to that of the latter, with mean value was 1.57. The maximum niche overlap value in the northern mountainous area was 5.78 and the minimum value was 4.74, which was the area with the highest mean value (5.22). The central region has the area with the lowest mean niche overlap value (4.77).

**Table 7:** Distribution table of dominant taxa along gradient niche breadth and overlapping space of water quality chemical index.

River	Section	District	Niche breadth	Niche overlap
Dongsha river	J1	Southern coastal area	1.79	4.70
	J2		1.60	5.09
Yinma river	J3		1.70	5.01
	J4		1.61	5.51
Xiyang river	J5	Central region	1.54	4.54
	J6		1.67	5.31
Qinglong river	J7		1.30	3.97
	J8	Northern mountain area	1.75	4.74
	J9		1.69	5.78
Daihe river	J10	Central region	1.77	5.24
	J11	Eastern coastal area	1.43	4.71
Xinhe river	J12		1.67	4.61
Shihe river	J13		1.54	5.09
	J14		1.58	5.68

In summary, water quality physical and chemical indexes have a certain extent effect on niche breadth and niche overlap of dominant taxa in different regions. The average niche breadth of the dominant taxa in the northern mountainous area was the highest along the gradient of water quality physical indexes, and the average niche breadth in the southern coastal area was slightly higher than that in other areas. The average niche breadth of the dominant taxa along the water quality chemical index gradient was lower in the northern mountainous area and higher in the southern coastal area. For niche overlap, the dominant taxa in the northern mountainous area showed higher values along all water quality index gradients, while the dominant taxa in the southern coastal area showed slightly higher overlapping values along the water quality chemical index gradients.

### Key driving factors of niche spatial differentiation of dominant taxa

#### Correlation degree between dominant taxa and water quality index

**Fig. 5:**
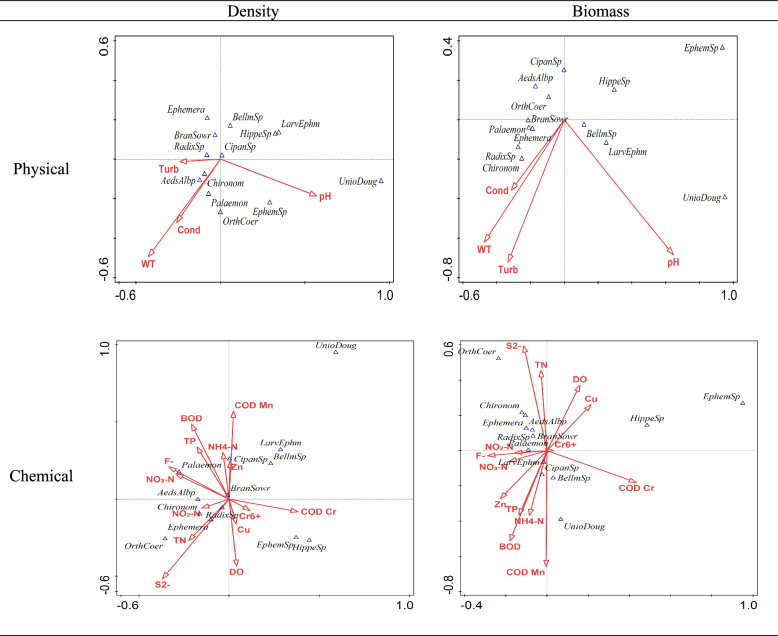
Results of CCA analysis of dominant taxa and water quality index.

The correlation between dominant species and physical and chemical indicators of water quality represents the extent to which habitat factors affect the density and biomass of dominant species. The CCA results were shown in Fig. 5.

In Fig. 5, WT and pH had the strongest impact on dominant species density, whereas WT, pH, and Turb most significantly affected biomass. For the chemical indicators of water quality, S^2-^, COD_Mn, COD_Cr, BOD had greater effects on the density and biomass of dominant species. DO had greater effects on density, while TN had greater effects on biomass.

#### Key driving factors of niche spatial differentiation of dominant taxa

For the physical and chemical factors of water quality that have a significant impact on dominant taxa, the calculation results of the partial correlation coefficients between niche and habitat factors of dominant taxa were shown in Table 8.

**Table 8:** Correlation degree between niche and habitat factors of dominant taxa.

Habitat environment	Index	Niche breadth correlation degree	Niche overlap correlation degree
Physical	pH	0.90	0.85
	WT	0.71	1.05
	Turb	0.76	0.82
Chemical	DO	0.88	0.71
	COD_Mn	0.69	0.82
	COD_Cr	0.95	1.18
	TN	0.86	0.75
	BOD	0.90	0.64
	S^2-^	0.83	0.98

In Table 8, among the physical and chemical indexes of water quality, the correlation between COD_Cr and the niche breadth of dominant taxa was the highest (0.95), while the correlation between COD_Mn and the niche breadth of dominant taxa was the lowest (0.69). In the water quality index, COD_Cr had the highest overlap correlation with the dominant taxa niche (1.18), followed by WT (1.05), and the correlation between the two was larger, while the overlap correlation between BOD and the dominant taxa niche was the lowest (0.64).

In conclusion, COD_Cr and BOD were the key driving factors for the spatial differentiation of niche breadth of dominant taxa. COD_Cr and WT were the key driving factors for the spatial differentiation of niche overlap of dominant taxa.

## Discussion

### Similarities and differences in dominant taxa

In this paper, the dominance model was used to select the taxa that could determine the overall behavior of the benthic macroinvertebrate community in Qinhuangdao, and 13 dominant taxa were obtained, among which *Unio douglasiae* and *Orthetrum coerulescens* were the most dominant. Gradient analysis of dominance revealed that the benthic macroinvertebrate community showed a significant hierarchical structure. The phylum *Mollusca* has *Unio douglasiae* as the core dominant taxa, with a biomass (350.98 g/m^2^) that far exceeds that of the other taxa, suggesting its potential role as a key Ecosystem Engineer. Yu^49^ conducted a study on the taxa diversity and distribution of freshwater bivalves in the Yangtze River Watershed (Anhui region) of China, and the results showed that: based on taxa occurrence rate and Berger-Parker dominance index, the dominant taxa in the study area at the present stage is *Unio douglasiae*, which is consistent with our research results. In eutrophic waters, high concentrations of suspended particles may further amplify their growth advantage^50^. The excessive nitrogen levels in many rivers in Qinhuangdao have stimulated phytoplankton proliferation, providing an extraordinary supply of bait for *Unio douglasiae*. The shell calcification process not only contributes directly to biomass accumulation, but also converts suspended particulate organic matter into sedimentary carbon through fecal excretion, creating a “biological pump” effect^51^. *Unio douglasiae* has filter feeding effect in the water bottom, which increases its dominance in the ecosystem^52^. *Orthetrum coerulescens* is highly adaptable and has strong predation ability in fresh water, and can inhabit various types of freshwater, including still water bodies (lakes, swamps), flowing water environments (streams, rivers), and artificial water areas (ditches, rice fields), so it has wide distribution and influence in the ecosystem^53,54^. In contrast, *Orthettrum coerulescens* and *Larva Ephemeropterae* have lower biomass, but as top predators (*Odonata*), their presence regulates community composition through a top-down effect, which is functionally complementary to the bottom-up control of mollusks’ upward resources^55^. The particularity of the current benthic community in Qinhuangdao resides in the fact that the unusual dominance of mollusks may imply ecological vulnerability. The adaptive expansion of *Unio douglasiae* to nitrogen pollution improves the water purification capacity in the short term, but population homogenization exacerbates the decline of system resilience, and in the event of disease outbreaks or environmental mutations, the water ecosystem may be drastically shaken.

### Niche breadth and niche overlap

The results showed that the Cond had a more significant effect on the distribution and niche breadth of the dominant benthic macroinvertebrate, while the effect of pH was relatively small. Elevated Turb increased niche overlap by homogenizing resource distribution, intensifying interspecific competition. Conversely, WT gradients reduced niche overlap through resource partitioning, which diminished competitive interactions among dominant taxa. Niche breadth is an important characterization of a taxa’ breadth of use of environmental resources and adaptive capacity, and its value directly reflects the competitive advantage of taxa in heterogeneous habitats^56^. In this study, we showed that the niche breadth of dominant benthic taxa in Qinhuangdao showed significant divergence, with *Chironomus* and *Radix* sp. having wider niches in Cond and Turb, indicating their strong adaptability to salinity fluctuations and turbidity changes. However, most taxa such as *Unio douglasiae*, *Orthetrum coerulescens*, and *Monopylephorus* sp. had mean values of niche breadth below 1.5, and *Unio douglasiae* in particular had the lowest value (1.00) for all physical metrics, suggesting a high degree of specialization in resource use. This narrow niche characteristic is similar to the benthic community in the Central Station Black-billed Grouse Reserve^24^. It suggests that under high-intensity human disturbance in Qinhuangdao, dominant taxa may lose their competitive advantages due to environmental threshold breakthroughs, triggering the risk of community succession. The niche overlap values then reveal the potential intensity of resource competition between taxa^56^.

Studies have shown that when the overlap value between taxa exceeds 4.0, the convergence of their ecological functions may threaten system stability^57^. In this study, the niche overlap values of *Ephemera* and *Aedes albopictus* under the influence of Turb were as high as 6.41, and the niche overlap values of *Aedes albopictus* on Cond and pH were all greater than 5.9, indicating that the two have a highly overlapping resource demand for high turbidity and high conductivity environments. In contrast, *Hippeutis* sp. (mean 2.99) and *Orthetrum coerulescens* (mean 5.04) had lower overlap values, and *Orthetrum coerulescens* had an overlap value of only 2.30 on the WT metrics, reflecting its mitigation of competitive pressures through niche differentiation. The vulnerability of narrow niche taxa may be exacerbated by environmental perturbations, despite their current biomass dominance. However, competitive stalemates with high niche overlap need to be mitigated by habitat heterogeneity restoration.

The niche characteristics and environmental adaptability of dominant taxa are influenced by water quality fluctuations. Large fluctuation degree of BOD promoted the taxa a wider niche to the change of organic material in water, and the small fluctuation of Cr⁶⁺ result in a narrow niche for taxa that have relatively little need to adapt to changes in heavy metals. The dominant taxa were more sensitive to changes in DO, resulting in lower niche overlap and too small changes in Cr⁶⁺. These dominant taxa were able to take advantage of such similar niche needs to optimize resource use, they coexist more easily and niche overlap will become higher. This suggests that heavy metal pollution forces taxa to congregate in a limited number of low-pollution microenvironments by compressing habitat suitability^58^. The niche breadths of *Unio douglasiae* and *Hippeutis* sp. under Cr⁶⁺ stress were 1.00 for both, whereas the overlap value of the two was as high as 5.00, suggesting that their survival spaces were highly overlapping and competitive. Janiga, et al.^59^ carried out a differential study on synergistic accumulation of toxic elements in benthic taxa in Zhongar-Alatua natural park, Kazakhstan, and the results showed that Cr⁶⁺ had the highest content in benthic macroinvertebrate, and its toxicity was stronger, which limited the utilization of resources by each taxa, which was consistent with our research results. The positive effect of BOD on niche breadth was the most significant (mean value 2.02), suggesting that organically contaminated environments contribute to the broadening of taxa’ resource use by increasing the abundance of degradable organic matter. *Chironomus* sp. and *Aedes albopictus* had niche breadths of 3.64 and 3.26, respectively, in the BOD gradient, suggesting that they are associated with strong adaptations to eutrophic water bodies. Moreover, such environments are usually accompanied by the accumulation of algal detritus, which provides an adequate food source for the larvae of Aedes aegypti^60^. The gradient distribution of DO, the factor with the lowest overlap (3.05), provides a key environmental substrate for taxa differentiation. Oxygen-demanding taxa *Ephemera* was concentrated in the high DO zone, while hypoxia-tolerant taxa *Monopylephorus* sp. dominated in the hypoxic zone, with a niche overlap value of only 1.26 between the two. It was shown that differences in DO were effective in alleviating interspecific competitive pressures, especially in localized hypoxic zones caused by inputs of aquaculture wastewater. Differences in DO were essential for maintaining community stability^61^.

In general, large BOD fluctuations represent large changes in the concentration of organic matter in the water, which is an important source of nutrients for benthic macroinvertebrates, so they often need to adjust niche to adapt to changes^62,63^. Relatively speaking, as an indicator of harmful metals, the small fluctuation of Cr⁶⁺ means that the concentration of harmful metals in the water is relatively stable, so taxa do not need to make too much niche adjustment to adapt to the environment^13,64^.

### Rationality analysis of key driving factors

The results showed that water environment characteristics of different regions profoundly influence the ecological adaptation strategies of dominant taxa and the resource competition pattern among taxa. Taxa in the northern mountainous areas were better at adapting to changes in the physical environment but more sensitive in the chemical environment, while those along the southern coastal area more were more adaptable to the chemical environment. Taxa screened out in environments with niche overlap, harshness or homogeneity have similar ecological strategies, and those along the southern coast also have certain competition in the utilization of physical resources, but the differentiation in chemical resources is more obvious. Rivers in mountainous areas are often characterized by strong hydrological heterogeneity, such as steep topography leading to significant flow gradients and diverse substrate types^65^. At the same time, they are less disturbed by humans, and the physical parameters of water quality are characterized by natural fluctuations. Habitat change prompts benthic macroinvertebrates to broaden their niche breadth^66^. Chi, et al.^67^ carried out a study on water-mediated macroinvertebrate community variation and niche differentiation in Shandong, China. The results showed that the average niche of dominant taxa in mountainous rivers was higher, and the niche breadth of benthic macroinvertebrate was smaller, which was consistent with our study results. The greater niche overlap in the northern mountains is due to the hardening of riverbanks, which weakens the natural hydrological gradient and forces taxa to compete for limited gravel attachment sites^68^. And the better the water quality in mountainous areas, the higher the niche overlap between them with a high degree of overlap of resources available to taxa in clean water bodies. The coast is subject to high-intensity human activity, and changes in niche breadth and overlap are less influenced by topography. Carcedo, et al.^69^ conducted a study on niche breadth and overlap of benthic invertebrates in the surf area in Argentina, and the results showed that the driving factors of niche change of benthic macroinvertebrate in the surf area were organic carbon and chlorophyll-α. The main reason for this is that surf areas are affected by tides and have different hydrodynamic characteristics than areas such as inland rivers, which leads to different responses of benthic macroinvertebrate to niche drivers^70,71^. In estuarine, beach and other areas, the key drivers of benthic animal niche may be organic carbon and chlorophyll-α.

COD_Cr, BOD and WT were the key drivers of niche breadth and overlap space differentiation of dominant taxa. COD_Cr reflects the pollution degree of difficult-to-degrade organic matter in the water body, and its value directly affects the detoxification metabolic pressure and resource availability of benthic animals^72^. The filter-feeding efficiency of the dominant taxon, *Unio douglasiae* will decrease in high COD_Cr conditions, which forces it to contract niche breadth. Yang, et al.^73^ reported wide niche breadth and large overlap for COD_Cr and WT among benthic macroinvertebrates in Jinan, China, while Liu, et al.^74^ highlighted the dominant influence of COD_Cr on macroinvertebrate abundance in response to urbanization in Shenzhen’s subtropical rivers. Both findings are consistent with our research results regarding the significant role of these water quality parameters. BOD refers to the amount of dissolved oxygen consumed by microorganisms to decompose organic matter in water, and its value reflects the intensity of organic pollution and the self-purification burden of the water body^72^. High BOD environments provide large amounts of heterogenous organic detritus such as algal detritus and cultured feeds, allowing taxa to evolve multiple substrate utilization capabilities. The increased niche breadth is premised on the fact that the large amount of food also attracts multiple taxa to concentrate on the same type of resource, while also increasing niche overlap. *Aedes albopictus* exhibited the highest niche breadth along the BOD gradient, demonstrating strong adaptability to organic matter resources and a high capacity to resolve and utilize nutrients in emission-rich environments. Conversely, its niche breadth was lowest along the Cr⁶⁺ gradient, suggesting severely constrained survival and reproductive capacity under harmful substance exposure. WT directly affected the physiological and ecological processes of benthic animals, with elevated temperatures typically promoting metabolic rates, growth, and reproduction. Despite the relatively low WT in the northern mountainous area, this region paradoxically exhibited the highest mean niche overlap among all regions and indicator combinations. Resource availability or interspecific competitive pressure limits the actual niche differentiation of species, resulting in a high degree of overlap. In general, high concentrations of COD-Cr and BOD can change the competition and food chain structure among benthic macroinvertebrate, thus affecting the niche breadth and overlap of benthic communities^75,76^. WT has a direct impact on the physiological and ecological processes of benthic macroinvertebrate, and at higher WT, the metabolic rate of benthic macroinvertebrate increases, promoting growth and reproduction^69,77^.

## Conclusions

The study aimed to identify dominant benthic macroinvertebrate taxa in Qinhuangdao, analyze the spatial differentiation of their niche breadth and overlap, and determine key driving factors. The main conclusions are as follows:

1. We successfully identified 13 dominant species, *Unio douglasiae* and *Orthetrum coerulescens* exhibited the highest dominance. Niche breadth and overlap analyses revealed significant spatial differentiation driven by water quality parameters, COD_Cr and BOD were the key driving factors for the spatial differentiation of niche breadth of dominant taxa. COD_Cr and WT were the key driving factors for the spatial differentiation of niche overlap of dominant taxa.
2. Water quality indicators profoundly influenced niche patterns, Cond and Turb promoted niche breadth, while Cr⁶⁺ compressed niche space and intensified interspecific competition. Regional heterogeneity played a critical role, northern mountainous areas showed higher niche breadth under physical water quality metrics but lower breadth with higher overlap under chemical metrics, whereas southern coastal areas exhibited expanded niche breadth along chemical gradients due to organic pollution.
3. Controlling organic pollution remains a critical and urgent priority. Elevated concentrations of COD_Cr and BOD can reduce niche breadth, intensify interspecific competition, and undermine community resilience. Therefore, it is essential to reduce the emissions of organic pollutants and heavy metals in order to preserve niche diversity and prevent competitive exclusion. Moreover, maintaining hydrological heterogeneity plays a vital role in supporting biodiversity. The relatively broader niche breadth observed in northern mountainous regions can be attributed to their natural hydrological gradients. Restoring river connectivity and substrate diversity in degraded areas such as urbanized coastal zones, can serve as effective measures to mitigate competitive pressures. Additionally, monitoring and managing fluctuations in water temperature, particularly in the context of ongoing climate change, are crucial for sustaining the physiological functions of benthic macroinvertebrates.
4. This study lacks in-depth exploration of niche drivers in estuarine and sandy beach ecosystems, where organic carbon and chlorophyll-α may play pivotal roles. Long-term effects of warming WT on metabolic stress and niche contraction in endemic species (e.g., *Unio douglasiae*) remain unquantified. Standardized monitoring integrating eDNA and niche modeling is recommended for dynamic brackish-freshwater transition zones, where current data are sparse.

This work provides a scientific basis for benthic habitat restoration in Qinhuangdao and a template for global basins facing synergistic pressures from urbanization and climate change.

## Data Availability

The data that support the findings of this study are available on request from the corresponding author C.H. upon reasonable request.
